# Massive Lumbosacral Subcutaneous Exudate After Surgical Treatment of a Large Lipomyelocele: Case Report and Literature Review

**DOI:** 10.1097/MD.0000000000001676

**Published:** 2015-10-02

**Authors:** Jun Gao, Xiangyi Kong, Yi Yang, Wenbin Ma, Renzhi Wang, Yongning Li

**Affiliations:** From the Department of Neurosurgery, Peking Union Medical College Hospital, Chinese Academy of Medical Sciences, Beijing, People's Republic of China.

## Abstract

Lipomyelocele is an uncommon type of lipoma that occurs with spina bifida. We present the clinical course and therapeutic process of a female who underwent resection of a lipomyelocele and developed a massive lumbosacral subcutaneous exudate postoperatively. The therapeutic process is described in detail, and a review of the relevant literature on lipomyelocele is presented.

A 23-year-old woman presented to our institution complaining of a large lumbosacral subcutaneous mass. She underwent surgical resection of the mass and untethering of the spinal cord under intraoperative neurophysiologic monitoring. A massive lumbosacral subcutaneous exudate developed postoperatively. After excluding cerebrospinal fluid (CSF) leakage, we placed a suction drain. Written informed consent was obtained from the patient for publication of this case report and any accompanying images. A copy of the written consent is available for review by the editor of this journal. Because of this, there is no need to conduct special ethic review and the ethical approval is not necessary.

Postoperative pathologic examination confirmed the diagnosis of lipomyelocele. Continuation of the negative-pressure drain for 1 week yielded >1000 mL of fluid. The patient recovered well and developed no further subcutaneous exudate.

In a patient with massive lumbosacral subcutaneous exudate after surgical treatment of a large lipomyelocele, continuous negative-pressure drainage can be an effective treatment method after excluding CSF leakage.

## INTRODUCTION

A lipomyelocele is a fibrofatty tissue mass in the spinal column that extends posteriorly through a spina bifida (SB) defect (gap or opening in a malformed spine)^1^ and is located under the skin, attached to the spinal cord. Lipomyelocele is the most common type of occult SB and often occurs in the lowest part of the spine.^2^ Generally, the tethered spinal cord needs to be surgically untethered or released to prevent or minimize symptoms.^3,4^ We present the case of a 23-year-old woman with a large lumbosacral lipomyelocele, located both inside and outside the spinal canal, associated with occult SB and tethered spinal cord. After surgical treatment, the patient developed a persistent, massive subcutaneous exudate at the surgical site. After a series of attempts at treatment, we concluded that continuous negative pressure could be an effective therapy for this condition. Here we present the patient's clinical course and, because lipomyelocele is relatively rare and the postoperative complication of subcutaneous exudate is also uncommon, a review of the relevant literature.

## CASE REPORT

**FIGURE 1:**
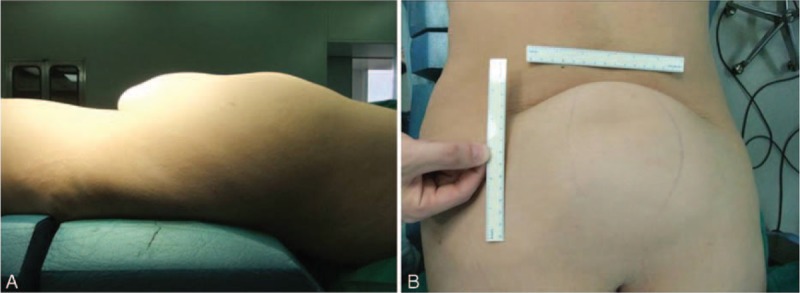
Preoperative photographs taken from the patient's (A) side and (B) back showing with lumbosacral lipoma with spina bifida. Although the mass was the size of a chicken egg originally, it was ∼20 × 15 × 10 cm at the time of the patient's admission to our hospital.

A 23-year-old woman was admitted to our hospital complaining of a large lumbosacral subcutaneous mass. She reported that when she was a child the mass had been the size of a chicken egg. It was soft and had grown slowly, without creating discomfort. However, after a natural parturition 3 years earlier, the mass had begun to grow much faster. At the time of the patient's presentation, the mass had grown to ∼20 × 15 × 10 cm. It was mobile and painless and had distinct borders (Fig. 1). The patient denied any motor, sensory, or excretory dysfunction. We did not identify any special circumstances in her medical history, family history, social history, or menstrual history related to her condition. Neurologic examination showed normal motor and sensory functions. Superficial and deep tendon reflexes were normal. Pathological signs and bilateral Lasègue sign were negative. No serum cancer-associated biomarkers were detected. Lumbosacral x-ray revealed physiologic spine curvature and normal intervertebral spaces, but the spinous process of the L5 vertebra was absent, suggesting a diagnosis of SB. On magnetic resonance imaging (MRI), the conus medullaris was observed to terminate at the L4 level, the L5 spinous process was absent and the vertebral plate had failed to close, and the cauda equina protruded from the spinal canal at the inferior edge of the L5 vertebral body. Massive adipose tissue with short T1 and long T2 signals was located both inside and outside the spinal canal, forming a large lumbosacral mass.

**FIGURE 2:**
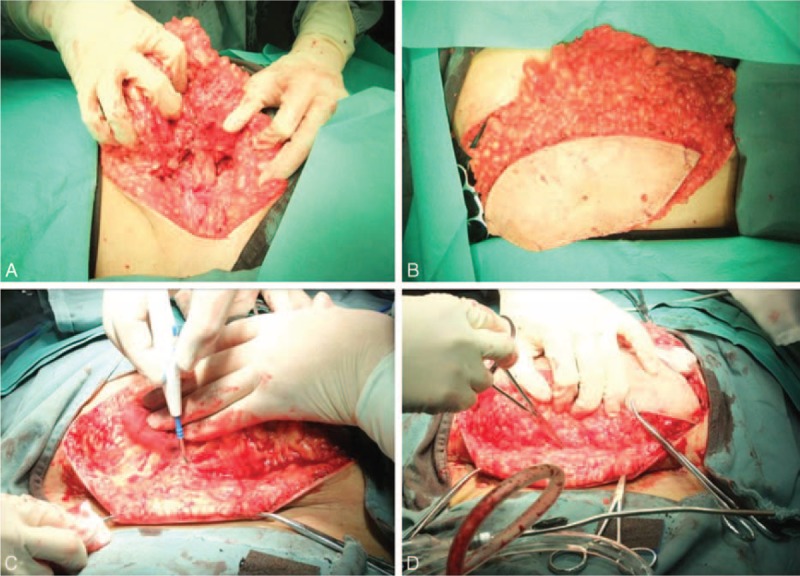
Intraoperative photographs showing surgical removal of the mass. Intraoperatively, the L5 spinous process was found to be maldeveloped, and the subcutaneous adipose tissue connected to the spinal canal through open laminae. Most of the mass, which weighed ∼1.1 kg, was removed.

The patient underwent surgery to resect the mass and untether the spinal cord (Fig. 2). After the lumbosacral musculature and fascia were exposed, we found a maldeveloped L5 spinous process and open laminae, through which the subcutaneous adipose tissue attached to the spinal canal. After removal of the L4 vertebral plate, a predominance of adipose tissue was exposed in the epidural space. The L3 vertebral plate was then removed and the cephalad end of the lipomyelocele was exposed. Most of the epidural adipose tissue, which weighed ∼1.1 kg, was removed. The epidural adipose tissue had invaded the intradural-extramedullary space at the inferior edge of the L4 vertebral body. After durotomy, we found the conus medullaris to be almost completely surrounded by adipose tissue, from which a large number of cauda equina nerves exited. To avoid nerve injury, the surgery was aided by intraoperative neurophysiologic monitoring (IONM) of motor-evoked potential (MEP). The areas of attachment between small nerve branches and the terminal filament were electrically stimulated. Only all the MEP amplitudes measured from the leg muscles were >100 μV, which we consider the criterion of MEP's response, did we perform the surgical procedures. Ultimately, only a portion of the intraspinal adipose tissue was removed. Pulsation of the spinal cord was restored. Watertight suturing of the spinal dura was performed, and a piece of artificial dura mater was placed. The open vertebral plate was repaired with a muscle flap. There was no CSF leakage and no active bleeding. A drainage tube was placed. Histopathologic examination confirmed the diagnosis of lipomyelocele.

**FIGURE 3:**
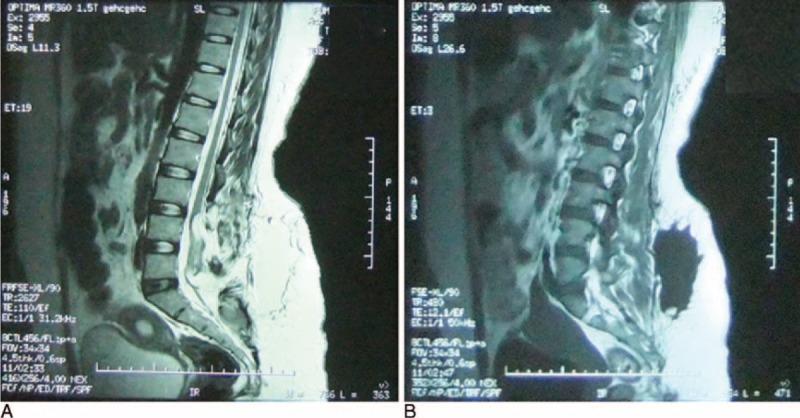
Preoperative MRI sagittal views of the lumbosacral region on postoperative day 6. A massive, irregular subcutaneous watery area with (A) long T2 and (B) long T1 signals at the L4 to S2 levels is observed. MRI = magnetic resonance imaging.

On the first postoperative day, 293 mL of blood-tinged fluid was collected from the drainage tube. Fluid volumes on the second and third days were 157 mL and 112 mL, respectively. No postoperative neurologic or urologic deterioration directly attributable to the surgery was observed, and the patient denied any major discomfort. The drainage tube was removed; however, 2 days after the removal of the tube, subcutaneous hydrops occurred and gradually increased. On postoperative day 6, the patient underwent lumbar MRI (Fig. 3), which revealed an irregular, watery area subcutaneously with long T1 and T2 signals at the L4 to S2 levels. Some short T1 and long T2 abnormalities were also observed inside the spinal canal (remnant lipomyelocele). We extracted a total of ∼200 to 300 mL of the subcutaneous fluid with a 50-mL syringe every day for a few days. However, there was no improvement in the patient's condition because the fluid formed continuously and the 2 lipid surfaces ventral and dorsal to the area of the fluid collection were separated by the exudate. To exclude CSF leakage, we tested the fluid sample for the presence of β-2 transferrin, and the result was negative. However, β-2 transferrin can only be detected in body fluids with at least 2.5% CSF contamination; therefore, CSF leakage into the fluid from fat liquefaction in this case could not be completely ruled out. A multidisciplinary discussion led to the conclusion that the chance of CSF leakage was slim because (1) the spinal dura had been sutured watertight during surgery; (2) the result of the β-2 transferrin test was negative, although its sensitivity might not reach 100% in fluids contaminated with only small amounts of CSF; (3) the patient did not develop postoperative positional headaches, a sign of CSF leakage; and (4) CSF net production would not be that fast. Instead, the massive subcutaneous exudate was more likely to be due to fat liquefaction and soft tissue exudation. The adipose tissue had not been completely removed and a somewhat damaged remnant was still in place, and it was hard for the 2 separated lipid surfaces, without rich blood supplies, to adhere to each other.^5^ This provided room for the accumulation of fat liquefaction, which in turn prevented healing, setting up a vicious circle. The key to treatment is therefore to break the circle, keeping the 2 lipid tissue surfaces closely apposed long enough for healing to occur.

**FIGURE 4:**
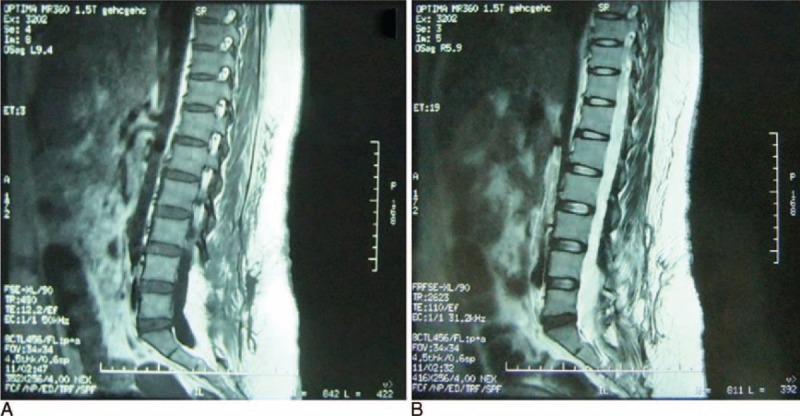
Postoperative MRI sagittal views of the lumbosacral region showing (A) ___ and (B) ___, with near disappearance of the watery area after 5 days of continuous suction drainage and 2 days of intermittent suction drainage. MRI = magnetic resonance imaging.

**FIGURE 5:**
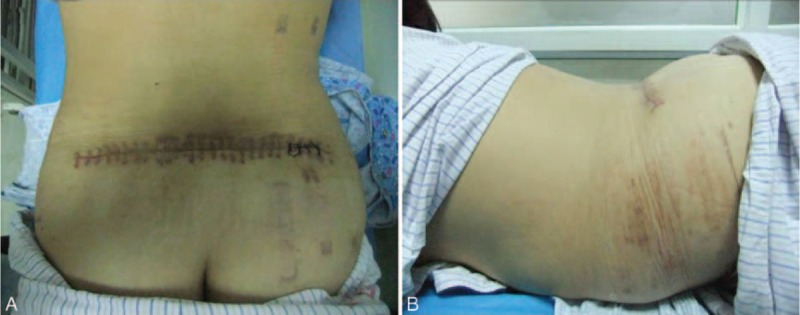
Photographs taken from the patient's (A) back and (B) side showing appearance of the operative site after treatment with suction drainage.

A suction tube was placed and the patient underwent continuous negative-pressure wound therapy. More than 1000 mL of light yellow and serosanguineous fluid was suctioned immediately. The cavity was closed and the cavity walls clamped tightly together. In the next 24 h, the suctioned fluid totaled <100 mL. On the third day, the fluid volume was very small. Continuous negative pressure was maintained for 5 days, at which point the vacuum aspirator was turned off intermittently for another 2 days. Meanwhile, we instructed the patient to get out of bed and do some activities.^6^ The patient recovered well and developed no further subcutaneous exudate. The suction tube was removed. Repeat MRI showed that the previous watery area had largely disappeared (Fig. 4). The appearance of the operative site after negative-pressure drainage is shown in Figure 5. The patient was followed for 6 months and continued to do well.

## DISCUSSION

Spina bifida is the most common neural tube defect and often occurs during the embryonic stage of development. Spina bifida can be divided into open and occult types. Open SB is also known as meningocele or myelomeningocele,^7^ whereas in occult SB cases, such as the present one, the neural tissue is unexposed and the skin is intact.^8^

Lipomyelocele is a type of lipoma that occurs with SB. The most common complaints include decreased leg strength, loss of bowel and bladder control, deformity of the legs or hips, back or leg pain, loss of reflexes and sensation in the legs, and abnormal curvature of the spine.^9^ There are often skin abnormalities, such as midline dimples, sinuses or tracts leading from the skin toward the spinal cord, birthmarks, fatty lumps, or small tufts of hair, overlying the tethered cord.

Conservative treatment with follow-up alone—operating only in neurologically worsening cases—is a therapeutic approach advocated by those who are concerned about postsurgical deterioration.^10^ However, with delayed surgery and the development of neurological deficits comes the risk of incomplete neurological recovery.^11^ Therefore, it is now commonly believed that surgery should be performed as soon as possible, before the occurrence or exacerbation of clinical symptoms.^12–14^ In addition, it has been demonstrated that surgery performed on symptom-free patients can prevent the development of late neurological deficits.^3,14^

The aim of surgery is to release the tethered cord and to rebuild the dural canal to prevent further fluid tethering.^15^ It should be noted that lipomyeloceles can be classified into 2 types by their anatomic relationships with the nerve roots.^15^ In type I, all nerve roots run superficially on the lipoma–cord interface, making it relatively easy to untether the cord. In type II, some nerve roots, originating from 1 or both sides, run within the lipomyelocele. In our case the conus medullaris was almost completely surrounded by adipose tissue, from which a large number of cauda equina nerves exited, making it impossible to remove the lipomyelocele completely. The most common surgical complications are nerve damage, neurologic deficits, and postsurgical deterioration.^16^ Hoving et al recommended that intraoperative determination of the necessary extent of surgical decompression be aided by IONM of MEP.^17^ Because the use of IONM can contribute to the safety of lipomyelocele surgery, we followed this recommendation. Although we conducted only subtotal resection, the absence of neurologic and urologic deterioration postoperatively illustrates the potential benefit of the use of IONM. However, the degree of resection is not directly related to postoperative clinical outcome. According to Klekamp et al,^18^ cases of near-complete neurological remission can be attributed to sufficient decompression by laminectomy and duraplasty without tumor resection.

In our case, the most challenging complication was the formation of massive subcutaneous exudate postoperatively. As mentioned, adipose tissue has a relatively poor blood supply, which makes it vulnerable to trauma or infection.^5^ During surgery, it is difficult to distinguish healthy from injured adipose tissue, so the injured tissue may not be completely resected, and the potential gap created by the resection makes liquefactive necrosis and exudate accumulation quite likely to occur.^5^ It should be emphasized here that despite watertight suturing, the large dural incision could have led to CSF leakage. Therefore, precise determination of the nature of the fluid is important in guiding treatment. A carbohydrate-free isoform of transferrin, β-2 transferrin is found almost exclusively in CSF. It is not found in blood, mucus, or tears and is thus a specific marker of CSF. Drainage fluid is presumed to be contaminated with CSF if β-2 transferrin is detected,^19^ which in our case it was not. This suggested that the CSF concentration, if any, was <2.5%. In addition, the patient did not develop positional headache, a sign of CSF leakage, postoperatively. Based on these facts, we made a somewhat bold decision to place a negative-pressure drain, which successfully maintained close apposition of the 2 lipid tissue surfaces until complete healing was achieved. We therefore believe that this method can be considered when treating massive lumbosacral subcutaneous exudate after lipomyelocele surgery.

From the overall view, even though the lipomyelocele is common, it is not common to have it in this size and probably this uncommon surgical complication of lumbosacral subcutaneous exudate is due to the size of the defect. In fact, in SB patients, this complication is mainly due to CSF leakage, especially when the closure of the dura is not sufficient. If the insufficient closure is not serious, the small transdural leakage is usually self-limiting, the subcutaneous exudate can spontaneously resorb, and the risk of infection or fistula is little. Nevertheless, if the layers are not intact, CSF will accumulate in the subcutaneous space, which itself can affect the healing. Moreover, this condition may be followed by co-infection, secondary fat liquefaction, and fibrinous exudate, although these could happen independently like our case. Generally, if the infection is confined to the extradural space, percutaneous cather drainage and adequate anti-infection treatment may help enough. If a subdural infection is confirmed, however, the suture or closure should be considered to be explored and the purulent material, if any, should be removed carefully and completely. The area should be sufficiently irrigated with antibiotic solution and water tightly sewed again. In our case, the subcutaneous exudate is too massive to be managed efficaciously by the traditional drainage method. Although it is not often necessary, we adopted the negative pressure, and it was proved to be a clever solution in this case with a good outcome.

Adipose tissue is traditionally thought to play a negative role in wound healing, a concept that has been challenged in recent years. Some new and special functions of adipose tissue have been discovered, including its secretory functions and its multi-differentiating potential as a source of adult stem cells. Some hormones, cytokines, and growth factors in adipose tissue have been found to contribute to metabolic regulation, energy balance, and homeostasis.^20^ Therefore, further study of the appropriate management of adipose tissue during lipomyelocele surgery should be performed.

## CONCLUSION

We have presented an uncommon case of massive lumbosacral subcutaneous exudate after surgical treatment of a large lipomyelocele. Continuous negative-pressure drainage may be an effective treatment for massive subcutaneous exudate after subtotal lipomyelocele resection after excluding CSF leakage.
